# Piezo1 protects against inflammatory bone loss via a unique Ca^2+^-independent mechanism in osteoclasts

**DOI:** 10.3389/fimmu.2025.1661538

**Published:** 2025-09-25

**Authors:** Satoru Shindo, Shin Nakamura, Avery Hawthorne, Alireza Heidari, Maria Rita Pastore, Motoki Okamoto, Maiko Suzuki, Manuel Salinas, Takumi Memida, Dmitriy Minond, Alexander Bontempo, Mark Cayabyab, Yingzi Yang, Janet L Crane, Maria Hernandez, Saynur Vardar, Patrick Hardigan, Xiaozhe Han, Steven Kaltman, Toshihisa Kawai

**Affiliations:** ^1^ Department of Oral Science and Translational Research, College of Dental Medicine, Nova Southeastern University, Fort Lauderdale, FL, United States; ^2^ Department of Engineering and Technology, College of Engineering and Computing, Nova Southeastern University, Fort Lauderdale, FL, United States; ^3^ Department of Pharmaceutical Sciences, Barry and Judy Silverman College of Pharmacy, Nova Southeastern University, Fort Lauderdale, FL, United States; ^4^ Cell Therapy Institute, Dr Kiran C. Patel College of Allopathic Medicine, Nova Southeastern University, Fort Lauderdale, FL, United States; ^5^ Department of Developmental Biology, Harvard School of Dental Medicine, Harvard Stem Cell Institute, Boston, MA, United States; ^6^ Department of Orthopedic Surgery, Johns Hopkins University School of Medicine, Baltimore, MD, United States; ^7^ Department of Pediatrics, Johns Hopkins University School of Medicine, Baltimore, MD, United States; ^8^ Department of Periodontology, College of Dental Medicine, Nova Southeastern University, Fort Lauderdale, FL, United States; ^9^ College of Health Sciences. University of Wyoming, WY, United States; ^10^ Department of Oral and Maxillofacial Surgery, College of Dental Medicine, Nova Southeastern University, Fort Lauderdale, FL, United States

**Keywords:** Piezo1, periodontitis, osteoclast, mechanosensing, PP2A, Akt

## Abstract

**Introduction:**

Bone integrity relies on mechanical stimulation, and its absence, such as in disuse osteoporosis or periodontitis, enhances osteoclast-mediated resorption. Although Piezo1 is a well-characterized mechanosensitive ion channel in several cell types, its function in osteoclast lineage cells has remained unclear.

**Methods:**

We examined Piezo1 expression and signaling in pre-osteoclasts (OCs) using mouse models of periodontal bone loss and in vitro differentiation assays. Genetic and pharmacological approaches were applied to manipulate Piezo1 activity. Downstream pathways were assessed with a focus on NFATc1 regulation, Akt phosphorylation, and PP2A activity. The therapeutic potential of the Piezo1 agonist Yoda1 was tested in inflammatory bone loss models.

**Results:**

Piezo1 was selectively expressed and functional in pre-OCs, where it acted as a mechanosensor to inhibit RANKL-induced OC-genesis. Activation of Piezo1 suppressed NFATc1 via a Ca^2+^-independent mechanism involving PP2A-mediated dephosphorylation of Akt, distinct from the canonical Ca^2+^-calcineurin pathway. In healthy periodontal bone, Piezo1 restrained osteoclast differentiation under mechanical loading, preserving bone mass. During periodontitis, reduced mechanical forces impaired Piezo1 function, resulting in unchecked osteoclast activation and pathological resorption. Pharmacological activation of Piezo1 with Yoda1 restored the anti-resorptive pathway and effectively prevented inflammatory bone loss, even in the absence of mechanical input.

**Discussion:**

Our findings redefine Piezo1 as a critical mechanosensor in pre-OCs and establish the Piezo1-PP2A-Akt axis as a novel regulator of NFATc1-driven OC-genesis. These results provide a mechanistic explanation for bone resorption in mechanically compromised states and highlight Piezo1 activation as a therapeutic strategy to mimic mechanical cues and suppress pathological OC-genesis in conditions such as periodontitis, rheumatoid arthritis, and osteoporosis.

## 1 Introduction

The onset and progression of periodontitis result from overactivation of the host immune response against opportunistic pathogens in the periodontal microbiome. This, in turn, leads to tissue-destructive inflammation of the periodontium, including alveolar bone resorption (1–3). Inflammation in the periodontium triggers angiogenesis, vasodilation, and increased vascular permeability, facilitating the enhanced migration of immune cells, including monocytes and macrophages, in response to T lymphocytes (4). Osteoclasts (OCs), monocyte lineage cells, play a key role in bone resorption in inflammatory bone-lytic diseases, such as rheumatoid arthritis (5, 6) and periodontitis (7, 8). Preosteoclasts (pre-OCs) are tartrate-resistant acid phosphatase (TRAP)^+^ mononucleated monocyte-linage cells which fuse to form multinucleated mature TRAP^+^ OCs (9) by activation of the master transcription factor (TF), Nuclear factor of activated T-cells cytoplasmic 1 (NFATc1) in a Receptor activator of nuclear factor kappa-b ligand (RANKL)-dependent manner (10). RANKL-mediated OC-genesis requires permissive costimulatory signaling from immunoreceptor tyrosine-based activation motif (ITAM) receptors (signaling transducers), such as Osteoclast-associated receptor (*OSCAR*) and Triggering receptor expressed on myeloid cells 2 (TREM2), to elicit the calcineurin/calmodulin signaling axis via Ca^2+^ oscillation (11, 12), in turn, upregulating NFATc1 expression.

Monocytes in the circulation, a significant source of pre-OCs (13–16), adhere to capillary endothelium and migrate into the homeostatic bone remodeling site as well as bone lytic lesions upon receiving signals from lipid mediators and specific chemokines, including Sphingosine 1-phosphate, monocyte chemoattractant protein-1 (MCP-1), C-X-C motif chemokine 12 (CXCL12), stromal cell-derived factor-1 (SDF-1), C-X3-C motif ligand 1 (CX3CL1) or macrophage migration inhibitory factor (MIF) (17–22). Intercellular adhesion molecule 1 (ICAM-1) expressed in endothelial cells (ECs) is a cell surface glycoprotein that regulates the migration of pre-OCs during periodontal inflammation (23, 24). Blood flow acts as a mechanical force on blood cells, such as T lymphocytes and platelets, as well as pre-OCs (25–28). Even though chronic inflammation in periodontitis causes expansion of capillary diameter (29), blood velocity in the vasculature of periodontitis lesions is significantly diminished (30). It is also reported that the rate of blood flow in healthy gingival tissue is significantly lower in the elderly than that of younger groups (31). Based on these lines of evidence, it can be posited that hematopoietic cells, including pre-osteoclasts (pre-OCs), in the periodontium affected by periodontitis, are subjected to reduced mechanical stress compared to those in healthy periodontal tissue. Increased mechanical stimuli can alter various activities of cells in the periodontium, including periodontal ligament cells and osteoblasts (32, 33), outcomes of the opposite condition whereby the periodontium receives less mechanical stress, especially alveolar bone, are largely unknown in the context of periodontitis.

Therefore, to shed more light on these unresolved questions, we turned to the mechanosensory system, the importance of which was highlighted by the 2021 Nobel Prize awarded for the discovery of mechanosensitive Piezo Ca^2+^ channels (34). Specifically, upon mechanical stimulation, the Piezo1 channel is opened by rearrangement of cytoskeletal actin anchored to the proximal of Piezo1, thereby eliciting Ca^2+^ influx which induces cell signaling for a variety of activities (35–42). Although a mouse model of Piezo1 conditional knockout in OC lineage cells showed no bone phenotype (43), *in vitro* results did show that shear stress can suppress OC-genesis (44, 45). Therefore, we were confident in hypothesizing that Piezo1 expressed on OC lineage cells is associated with the pathogenic bone phenotype. Here, we report that Piezo1 is the major mechanosensory receptor expressed on pre-OCs and that diminished mechanostress to pre-OCs can impede the Piezo1-mediated downmodulation of OC-genesis in periodontitis without affecting the magnitude of local inflammatory mediators. We further discovered that the activation of Piezo1 expressed on pre-OCs elicits a unique cell signaling axis involving PP2A-mediated dephosphorylation of Akt which, in turn, suppresses the expression of NFATc1, a master TF for RANKL-induced OC-genesis.

## 2 Materials and methods

### 2.1 Animals

To generate Piezo1^fl/fl^ LysM-Cre (Piezo1^ΔLysM^) mice, Piezo1^fl/fl^ mice (Jackson Laboratories, stock no. 029213) mice were crossed with LysM-Cre mice (Jackson Laboratories, stock no. 004781). Piezo1^fl/fl^ littermates were used as controls. Genotypes were confirmed by PCR from tail DNA. Wild-type C57BL/6J mice (Jackson Laboratories) were used in experiments not requiring genetic manipulation. 6- to 8-week-old males were used. The experimental procedures employed in this study were approved by the NSU IACUC (Protocol #TK7).

### 2.2 Cell culture

Bone marrow-derived mononuclear cells (BMMCs) were collected from tibias and femurs of mice and were cultured with minimum essential medium-α (MEM-α) supplemented with 10% fetal bovine serum (FBS), streptomycin (100 µg/mL), penicillin (100 U/mL), Amphotericin B (0.25 µg/mL), L-glutamine (292 μg/mL) and M-CSF (25 ng/mL; BioLegend, San Diego, CA, USA) at 37 °C in humidified air with 5% CO_2_ for 3 days to obtain pre-OCs. Subsequently, pre-OCs were differentiated to OCs using RANKL (10 ng/mL; BioLegend). Human Peripheral Blood Mononuclear Cells (PBMCs) were purchased from STEMCELL Technologies (Vancouver, Canada). PBMCs were stimulated with M-CSF (30 ng/mL; BioLegend) at 37 °C in humidified air with 5% CO_2_ for 3 days. Human pre-OCs were differentiated to OCs using RANKL (100 ng/mL; BioLegend). Yoda1 (Tocris), GsMTx4 (Tocris), LY294002 (Cell Signaling Technology), FK506 (Tocris), Ocadaic acid (R&D Systems) and DT-061 (Tocris) were administered with validated concentration. Aa vehicle control, the same concentration of Dimethylsulfoxide (DMSO, Sigma) was used.

### 2.3 Cell culture under flow or hydrostatic pressure

Shear stress was generated by a rocker (Thermo Fisher Scientific) or ibidi pump system (ibidi, Fitchburg, WI). Pre-OCs were seeded in wells of a 24-well plate (1x10^6^ cells/well) and then stimulated with shear stress by rocking (15°, 30 rpm). On the other hand, for the ibidi pump system, pre-OCs were cultured in the µ-Slide I 0.4 Luer (3x10^5^ cells/well). Cells were cultured with shear stress at 5 dyn/cm^2^ or 20 dyn/cm^2^. For hydrostatic pressure (HP) loading, cell culture dishes were positioned at the bottom of a beaker (1000 ml or 100 ml), and medium was added to heights of 5.5 cm, generating consistent HP. Control cells were cultured with a 1.2 cm medium height under atmospheric pressure (46).

### 2.4 Tartrate-resistant acid phosphatase staining

TRAP staining was performed with a TRAP staining kit (Sigma-Aldrich) according to the manufacturer’s protocol. Briefly, OCs were fixed by a citrate (0.38 mol/L)/acetone solution for 30 seconds at room temperature. Cells were stained with a TRAP staining solution (L(+)-tartrate buffer, 0.67 mol/L; acetate buffer, 2.5 mol/L; Naphthol AS-BI phosphoric acid, 12.5 mg/mL; and Fast Garnet GBC base, 7.0 mg/mL) for 10 min at 37°C in the dark. OCs with ≥ 3 nuclei were determined to be multinucleated OCs.

### 2.5 Pit formation assay

A plate coated with calcium phosphate was prepared according to previous reports (47, 48). Briefly, 0.12 M Na_2_HPO_4_ and 0.2 M CaCl_2_ (50 mM Tris-HCl, pH 7.4) were mixed at 37°C. The calcium phosphate slurry was washed with sterile water and then applied into wells of a culture plate and dried at 37°C overnight. BMMCs (3.0 × 10^5^ cells/well for a 96-well plate or 1× 10^6^ cells/well for a 24-well plate) were then seeded in wells of calcium phosphate-coated plates, respectively. After treating cells for 6 days, the plates were washed with 10% sodium hypochlorite for 10 min and then dried overnight. The pit areas were microscopically imaged (Evos Cell Imaging System, Thermo Fisher Scientific). Images were analyzed using ImageJ software (version 1.50).

### 2.6 Measurement of intracellular Ca^2+^ concentration

Cells (4×10^4^ cells/well) were seeded in wells of a black wall/clear bottom plate. Fluo-8 No Wash Calcium Assay Kit (AAT Bioquest, Pleasanton, CA) was employed to measure Ca^2+^ influx in accordance with the manufacturer’s protocol. Briefly, Fluo-8 NW and 0.04% Pluronic™ F-127 in HHBS buffer were added for 30 min at 37°C, followed by 30 min at room temperature. After Yoda1 treatment, fluorescence alternately excited at 490 nm and emission at 525 nm was measured every 10 sec using a FilterMax F5 Microplate Reader (Molecular Devices, San Jose, CA).

Ca^2+^ influx was also analyzed under flow conditions with the BioFlux One system (Fluxion Biosciences, Oakland, Ca) or ibidi pump system (ibidi). For the BioFlux One system, a 48-well microfluidic plate (Fluxion Biosciences) was first coated for 1 h at room temperature with rat tail type 1 collagen (50 μg/ml; Thermo Fisher Scientific) in 0.2% acetic acid. Before using the plate, microfluidic channels were washed with PBS, followed by the introduction of cells in the channels. After 24 h, the Fluo-8 No Wash Calcium Assay Kit was employed to image Ca^2+^ influx. The assay was performed with a wall shear stress at 20 dyn/cm^2^. Fluorescence intensity was measured and analyzed by the BioFlux One system (Fluxion Biosciences). For ibidi pump system, pre-OCs were seeded in µ-Slide I 0.4 Luer (ibidi) at 3x10^5^ cells/plate. The assay was performed with a wall shear stress at 20 dyn/cm^2^. Fluorescence intensity was measured and analyzed by the EVOS (Thermo Fisher Scientific).

### 2.7 siRNA transfection for knockdown of Piezo1.

BMMCs were seeded in wells of a 96-well plate (3×10^5^ cells/well) or 24-well plate (1×10^6^ cells/well). After 24 h, BMMCs were maintained in Opti-MEM™ Reduced Serum Medium (Thermo Fisher Scientific) and transfected with 50 nM Piezo1-specific siRNA (107969, Thermo Fisher Scientific; siPiezo1), PP2A-specific siRNA (152168, Thermo Fisher Scientific; siPP2A), PP2B-specific siRNA (162266, Thermo Fisher Scientific; siPP2B) or negative control siRNA (AM4611, Thermo Fisher Scientific; siCTL) using Lipofectamine™ RNAiMAX Transfection Reagent (Thermo Fisher Scientific) according to the manufacturer’s instructions. 48 h post- transfection, BMMCs were used for indicated experiments.

### 2.8 Quantitative polymerase chain reaction

Total RNA was extracted from pre-OCs using the PureLink RNA Mini Kit (Thermo Fisher Scientific), following the manufacturer’s protocol. The first strand cDNA was assembled from 100 ng of sample RNA using a Verso cDNA Synthesis Kit (Thermo Fisher Scientific). Amplification reactions were performed by Taqman Fast Advanced Master Mix (Thermo Fisher Scientific) or SYBR Green Master Mix (Thermo Fisher Scientific). The resultant cDNA was amplified by specific probes (Thermo Fisher Scientific) for *Gapdh* (Mm99999915_g1), *Piezo1* (Mm01241549_m1), *Piezo2* (Mm01265861_m1), *Trpa1* (Mm01227437_m1), *Trpv4* (Mm00499025_m1), *Stoml3* (Mm01289590_m1), *Kcnk10* (Hs01026663_m1), *Kcnk4* (Mm00434626_m1), *Kcnk1* (Mm00434624_m1), *Ocstamp* (Mm00512445_m1), *Mmp9* (Mm00442991_m1), *Ctsk* (Mm00484039_m1), *Acp5* (Mm00475698_m1), *Oscar* (Mm01338227_g1), *Nfatc1* (Mm00438670_m1), *Tnfsf11* (Mm00441906_m1) and *Tnfsf11b* (Mm00435454_m1) on a QuantStudio™ 3 (Thermo Fisher Scientific). The ratios of mRNA levels to those of the control gene were calculated using the ΔCt method (2^−ΔΔCt^).

### 2.9 PCR array

After extraction of total RNA from cells and synthesis of cDNA described above, the resultant cDNA was tested using Taqman Fast Advanced Master Mix (Thermo Fisher Scientific) and TaqMan^®^ Array Mouse Osteogenesis (Thermo Fisher Scientific) according to the manufacturer’s instruction. An integrated web-based software package was used for data analysis (https://www.thermofisher.com/account-center/simplified-username.html).

### 2.10 Phospho antibody array

The Phospho Explorer Antibody Array was used according to the manufacturer’s instruction (Full Moon BioSystems, Sunnyvale, CA) to profile the levels of phosphorylated proteins. Briefly, cell lysate from BMMCs was collected and quantified by BCA Protein Assay Kit (Thermo Fisher Scientific). Microarray slides were blocked, and proteins were labeled using biotin and then coupled to slides. Slides were washed, and Cy3-streptavidin was added to bind biotin. Fluorescence intensity was measured and analyzed by the manufacturer (Full Moon BioSystems). The clustering of target proteins and signaling pathways was assessed using Kyoto Encyclopedia of Genes and Genomes (KEGG) and Ingenuity Pathway Analysis (IPA) (Qiagen, Germantown, MD).

### 2.11 Western blotting

After incubation for various times, pre-OCs were lysed by incubation on ice for 30 min with RIPA buffer (Thermo Fisher Scientific) supplemented with a protease inhibitor cocktail (Sigma-Aldrich). Protein concentration of the resultant lysates was measured with the BCA Protein Assay Kit (Thermo Fisher Scientific). Fifteen μg of sample per lane were loaded onto a 4–12% sodium dodecyl sulfate–polyacrylamide gel electrophoresis (SDS-PAGE) gel (Thermo Fisher Scientific). Proteins separated in the SDS-PAGE gel were electrotransferred to a polyvinylidene difluoride membrane. The detection of specific proteins in pre-OCs was assessed using anti-phospho-Akt rabbit mAb (193H12, 1:1000; Cell Signaling Technology, Danvers, MA), anti-Akt rabbit mAb (C67E7, 1:1000; Cell Signaling Technology), anti-phospho-p38 MAPKs rabbit mAb (3D7, 1:1000; Cell Signaling Technology), anti-p38 MAPKs rabbit mAb (D13E1, 1:1000; Cell Signaling Technology), anti-phospho-ERK rabbit mAb (D13.14.4E, 1:2000; Cell Signaling Technology), anti-ERK rabbit mAb (137F5, 1:2000; Cell Signaling Technology), anti-phospho-JNK rabbit mAb (81E11​, 1:1000; Cell Signaling Technology), anti-JNK rabbit mAb (9258, 1:1000; Cell Signaling Technology), anti-IkBa rabbit mAb (L35A5, 1:1000; Cell Signaling Technology), anti-phospho-PP2A mouse mAb (F-8, 1:1000; Santa Cruz Biotechnology, Dallas, TX), anti-PP2A mouse mAb (6F9, 1:1000; Santa Cruz Biotechnology) or an anti-GAPDH rabbit mAb (14C10, Cell Signaling Technology). Protein bands that reacted with the respective antibody were visualized by incubation with an HRP-conjugated rabbit or mouse secondary antibody (Cell Signaling Technology), followed by detection using ECL Western Blotting Substrate (Thermo Fisher Scientific). Densitometric analysis was performed using ImageJ software (Version 1.50).

### 2.12 Co-immunoprecipitation assay

To examine the interaction between RANK and TRAF6 or Piezo1 and PP2A, Co-IP was performed. Pre-OCs were lysed in ice-cold RIPA buffer (Thermo Fisher Scientific) supplemented with a protease inhibitor cocktail (Sigma-Aldrich). The lysates were cleared by centrifugation and incubated overnight at 4°C with an anti-RANK antibody (sc-374360, 10 ug/ml/sample, Santa Cruz Biotechnology). Or anti-Piezo1 antibody (2-10, 10 ug/ml/sample, Thermo Fisher Scientific). Immune complexes were captured using Protein A/G magnetic beads (Thermo Fisher Scientific) for 1 h at RT with gentle rotation. Beads were washed three times with Tris-Buffered Saline with Tween 20 (TBST), and bound proteins were eluted in LDS sample buffer, followed by immunoblotting with anti-TRAF6 antibody (E2K9D, 1:1000; Cell Signaling Technology) or anti-PP2A antibody (F-8, 1:1000; Santa Cruz Biotechnology).

### 2.13 Immunofluorescence

Pre-OCs cultured on Millicell EZ SLIDE (Sigma) were fixed with 4% paraformaldehyde at room temperature for 10 min, permeabilized with 2% Triton X-100, and blocked with 5% BSA in PBS for 1 h. Cells were incubated with anti-Piezo1 antibody conjugated with Alexa Fluor^®^ 594 (CL9714​, 1 ug/ml, Novus, Centennial, CO), isotype control antibody conjugated with Alexa 594 (141945, 1 ug/ml, Novus), anti-NFATc1 antibody (7A6, 1 ug/ml, Santa Cruz Biotechnology) or isotype control antibody (MOPC-21​, 1 ug/ml, Bio X Cell) at 4°C overnight. Cy3-conjugated goat anti-mouse IgG secondary antibody (1:100, Jackson ImmunoResearch, West Grove, PA) was applied for 1 hour at room temperature, followed with CellMask™ Green Actin Tracking Stain (Thermo Fisher Scientific) and Fluoromount-G containing DAPI (Thermo Fisher Scientific). Immunofluorescence was imaged with a Zeiss LSM880 confocal microscope (Carl Zeiss, Jena, Germany).

### 2.14 Mouse model of periodontal disease

To induce periodontal disease in mice (6–8 weeks old; male or female), the maxillary second molar was attached with a 5–0 silk ligature, following the previously published protocol (49, 50). Yoda1 (0.4 mg/kg), DT-061 (0.4 mg/kg) or DMSO (0.86%) was diluted in PBS with 5% ethanol, followed by injection through the intraperitoneal route every 2 days (day 0, 2, 4 and 6). After 7 days, mice were euthanized for postmortem analyses.

### 2.15 Monitoring Blood Perfusion Unit (BPU)

**Figure 1:**
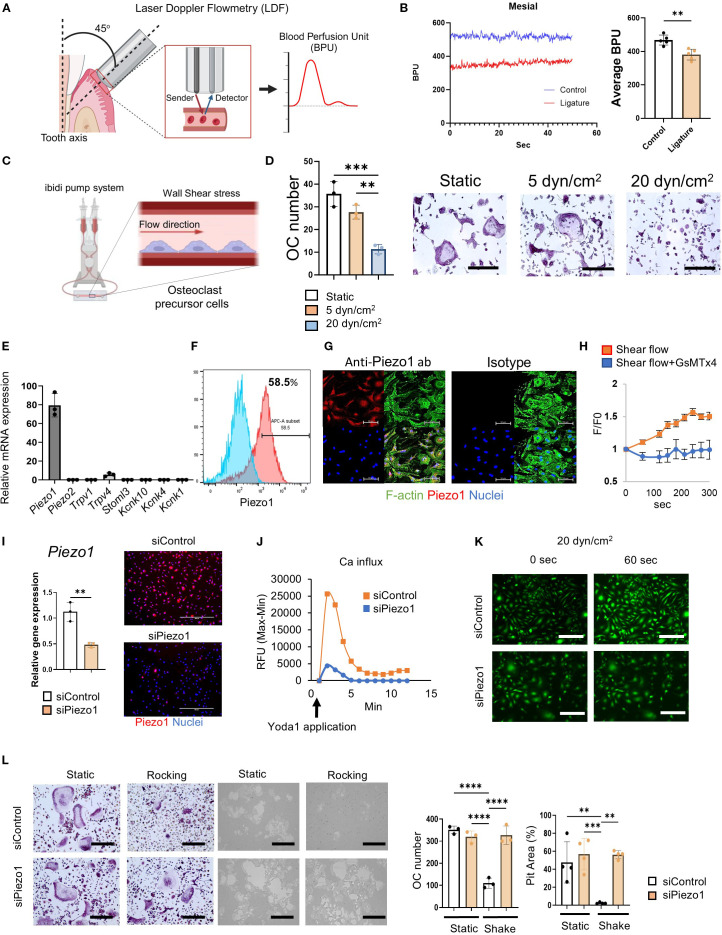
Shear stress inhibited OC-genesis through Piezo1. **(A, B)** Real-time BPU that reflects the flow rate of local vasculature in the palatal gingival tissue of a live mouse was measured by Laser Doppler Flowmetry as shown. BPU was measured at the mesial and distal sites of second maxillary molar with or without a silk ligature to induce periodontitis. Continuous detections of temporal change of real-time BPU (50 s) were displayed. **(C, D)** MCSF (25 ng/ml)-primed murine bone marrow-derived mononuclear cells were employed as pre-OCs. TRAP staining was conducted after 6 days of RANKL treatment. Scale bar: 10 μm **(E)** Expression of mechanosensory receptors, including *Piezo1*, *Piezo2, Trpv1, Trpv4, Stmol3, Kcnk10, Kcnk1* and *Kcnk4*, in pre-OCs was determined by qPCR. **(F, G)** Piezo1 protein expression was detected by flow cytometry and immunofluorescence, respectively. Scale bar: 50 μm **(H)** Fluo-8-treated pre-OCs with or without GsMTx4 (1 uM) were stimulated by shear flow at 20 dyn/cm^2^ using the Bioflux microfluidics system. Ca^2+^ influx was visualized and analyzed with the Bioflux system. **(I)** To silence *Piezo1* expression, Pre-OCs were transfected with siRNA specific to Piezo1 (siPiezo1). siPiezo1-mediated silencing efficacy was evaluated by qPCR and immunofluorescence staining in comparison to control siRNA treatment (siControl). **(J)** siPiezo1-mediated Piezo1 loss-of-function was determined by measuring Yoda1-enhanced Ca^2+^ influx. Fluo-8-treated pre-OCs were stimulated with Yoda1; subsequently, kinetic fluorescence intensity was measured every 10 sec by plate reader. **(K)** Fluo-8-treated pre-OCs in µ-Slide I 0.4 Luer were stimulated with 20 dyn/cm^2^ of shear stress generated by the ibidi pump system, followed by time-lapse images taken every 1sec. Representative image after 1 min of shear flow exposure was displayed. Scale bar: 100 μm **(L)** TRAP staining or pit formation assay were conducted after 6 days or 9 days of RANKL treatment, respectively. The number of TRAP-positive multinucleated OCs was measured, and measurement of pit area was performed. Scale bar: 10 μm. Data represent the mean ± SD of three independent experiments. *p < 0.05 **p < 0.01 ***p < 0.001 ****p < 0.0001.

Real-time BPU was monitored by Laser Doppler Flowmetry (OxyFlo pro, Oxford Optronix, UK). A fine needle-type blood flow probe (Diameter: 0.5 mm, Oxford Optronix) was attached to the mesial or distal surface of palatal gingiva, respectively (**Figure 1**). Real-time data were captured and analyzed by Labchart 8 software (ADInstruments, Colorado Springs, CO).

### 2.16 Histological analysis

Murine maxillary bones were fixed in 4% paraformaldehyde overnight at 4°C before decalcification in 10% EDTA at 4°C for 2 weeks. Tissues were embedded in an OCT compound (Sakura Finetek USA, Torrance, CA, USA) overnight at −20°C and cut into 8 μm sections with a cryostat (Leica Biosystems, Deer Park, IL). TRAP staining of decalcified periodontal tissue was performed using an Acid Phosphatase Leukocyte (TRAP) Kit (Sigma-Aldrich), as described above, followed by nuclear counterstaining with methyl green. Sections were imaged with an EVOS XL Core microscope (Thermo Fisher Scientific). For immunofluorescence-based detection of OC-STAMP and phospho-Akt, the sections were reacted with anti-OC-STAMP rabbit pAb (HPA031116, 1:200; Sigma-Aldrich) or anti-phospho-Akt rabbit mAb (D9E, 1:200; Cell Signaling Technology) as the primary antibody at 4°C overnight. Cy3-conjugated anti-rabbit IgG FC goat pAb (1:200; Jackson ImmunoResearch) was used as a secondary antibody. The stained sections were mounted with Fluoromount-G containing DAPI (Thermo Fisher Scientific). Immunofluorescence was observed with a Zeiss LSM880 confocal microscope (Carl Zeiss, Jena, Germany).

### 2.17 Micro-CT analysis

Mouse maxillary alveolar bone was fixed in 4% phosphate-buffered paraformaldehyde and stored at 4˚C for 16 hours. Micro-CT images were obtained with the Microfocus X-ray CT scanning system (Skyscan 1176, Bruker, Billerica, MA), using the following settings: acceleration voltage, 50 kV; current, 500 µA; voxel size, 18 µm/pixel; matrix size, 2,000 × 1,336. Images were reconstructed with NRecon software, version 1.7.0.3 (Bruker), and images of both ligature side and control untreated side were acquired. As regions of interest (ROI), 50 sliced images coronally from the contact point between the maxillary first molar and maxillary second molar were evaluated. Bone volume (BV) of the whole palatal alveolar bone, including the ipsilateral hard palate, was measured using three-dimensional (3D) analysis CTAn software, version v.1.18 (Bruker). 3-dimensional images were obtained using CTVox software, version 3.2.0 (Bruker). To evaluate periodontal bone resorption, distances from the cement–enamel junction to the alveolar bone crest on the palatal side of root were measured for the maxillary second molar.

### 2.18 Statistical analysis

Statistical analyses were performed by one-way ANOVA and Tukey’s Honestly Significant Difference (HSD) test to compare differences among multiple groups and Student’s t-test for comparisons between two groups. All statistical analyses were performed using GraphPad Prism, version 10.0.1 (GraphPad Software, Inc., La Jolla, California, USA). Statistical significance was considered to be at p < 0.05. All data were expressed as the mean ± SD.

## 3 Results

### 3.1 Blood flow was reduced in murine periodontitis

Although, as noted above, the blood flow rate in the vasculature of human periodontitis lesions is significantly diminished (30); however, it is unknown whether blood flow in the periodontitis induced in mice is also reduced. Therefore, to induce murine periodontitis, a silk ligature was attached to the second maxillary molar for 7 days. To assess the impact of periodontitis on the local blood flow, the blood perfusion unit (BPU) of gingival tissue was measured using Laser Doppler Flowmetry. Irrespective of inflammation induced in the periodontal tissue, the BPU in periodontally diseased tissue was significantly reduced compared to that in healthy tissue (**Figures 1A, B**). Since interstitial pressure is generally considered to be proportional to the local blood flow rate (51, 52), it is assumed that the mechanical stress to the cells in the laminar propria of periodontal tissue is also reduced.

### 3.2 Mechanical stress suppressed RANKL-induced OC-genesis *in vitro*

According to a previous report, the blood flow rate in the microcapillaries of healthy periodontal tissue is approximately 20 dyn/cm^2^, whereas that in diseased periodontal tissue is diminished to approximately 5 dyn/cm^2 30^. To compare the effect of flow rates, a microfluidics system was employed to evaluate the effects of fluid shear stress on RANKL-induced OC-genesis. OC-genesis, as well as OC-related gene expression (*Ocstamp*, *Mmp9*, *Acp5*, *Oscar*, *Dcstamp* and *Nfatc1*), were significantly suppressed by a high flow rate (20 dyn/cm^2^) compared to a low flow rate (5 dyn/cm^2^) or static condition (**Figures 1C, D**; **Supplementary Figure S1a**). However, Piezo1 expression did not significantly differ between high- or low-flow conditions and static control (**Supplementary Figure S1a**). Moreover, shear flow generated in the tissue culture plate by a rocker (15°, 30 rpm) also suppressed RANKL-induced OC-genesis (**Supplementary Figures S1b, c**). Hydrostatic pressure (HP) via the tissue interstitial fluid has an important role in providing mechano-stimulation to cells (53). To examine the effect of HP on OC differentiation, the OCs were cultured in two different culture flasks following the protocol published by another group (46, 54). A 5.5 cm deep beaker with 100 ml of medium gives approximately HP of 3.7 mmHg, compared to a 1.2 cm deep flask with 100 ml of medium (0 mmHg). Such a difference of HP promoted Piezo1 stimulation, which, in turn, significantly suppressed OC differentiation (**Supplementary Figure S1d**).

### 3.3 Piezo1 expressed on pre-OCs functioned as a mechanosensory Ca^2+^ channel

Out of 8 major mechanoreceptors (*Piezo1*, *Piezo2*, *Trpv1*, *Trpv4*, *Stoml3*, *Kcnk10*, *Kcnk4* and *Kcnk1*), Piezo1 mRNA was expressed at the highest level (**Figure 1E**). The protein expression of Piezo1 in pre-OCs was confirmed by both immunofluorescence staining and flow cytometry (58.5%) (**Figures 1F, G**). GsMTx4, a spider venom that selectively inhibits Piezo1 (55–57), suppressed Ca^2+^ influx induced in pre-OCs via microfluidics-generated shear flow (**Figure 1H**), indicating that pre-OCs appeared to sense shear flow-generated mechanical force via Piezo1.

### 3.4 Sensing of shear stress by Piezo1 expressed on pre-OCs suppressed *in vitro* OC-genesis

**Figure 2:**
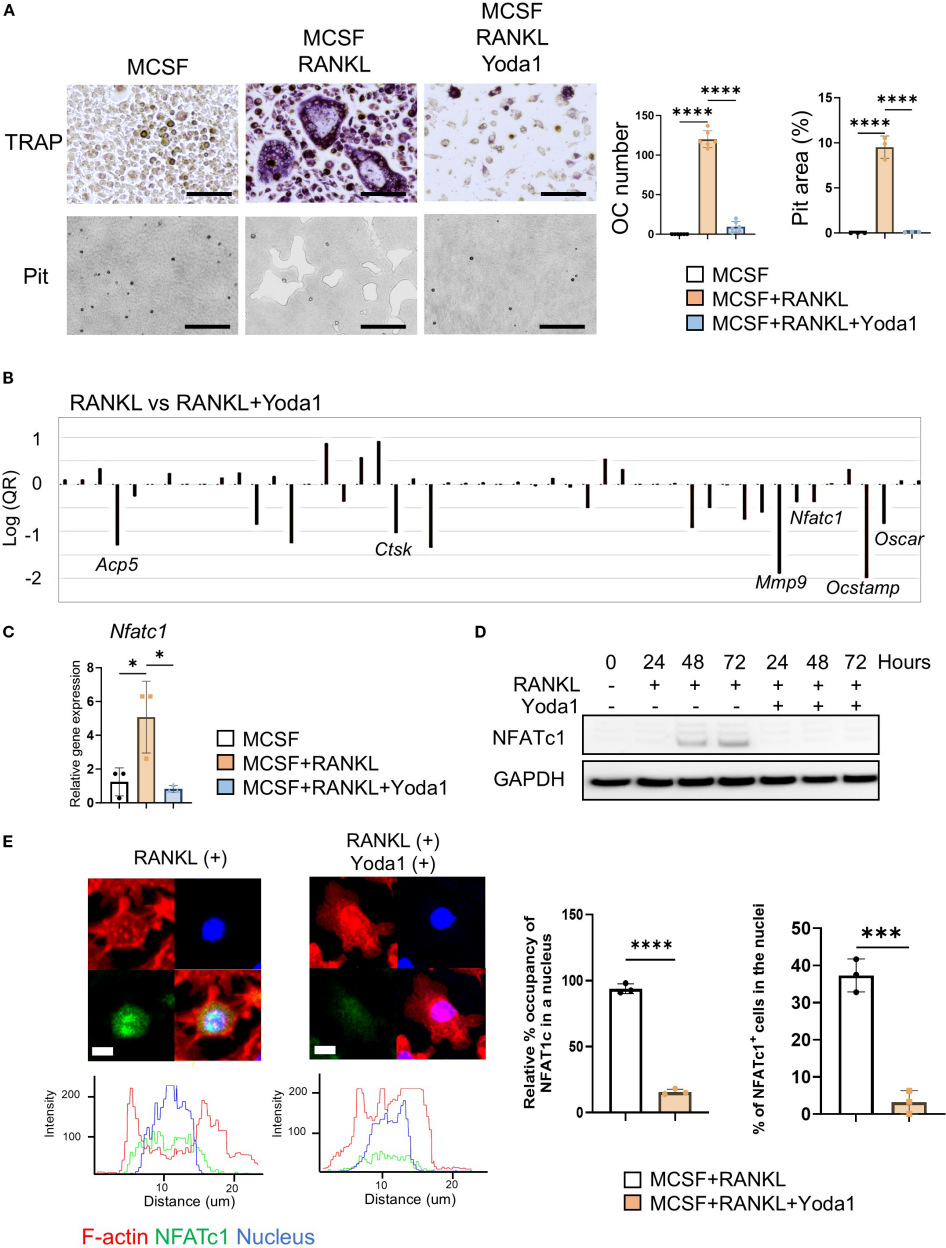
Pharmacological Piezo1 activator inhibited OC-genesis and function. **(A)** Pre-OCs were treated with RANKL (10 ng/ml) in the presence or absence of Yoda1 (5 µM) or vehicle control. After 6 days, TRAP-positive OCs with three or more nuclei were counted as mature OCs. Pit formation activity by OCs was evaluated by imaging and calculation using Image J (Version 1.50). Scale bar: 10 μm **(B)** PCR array was performed to identify osteoclast-related genes regulated by Yoda1. **(C, D)** NFATc1 mRNA and protein expression was determined from pre-OCs stimulated with or without Yoda1 at day 1, day 2 and day 3 by qPCR or Western blot analysis, respectively. GAPDH was loading control. Densitometric analysis of three independent experiments was performed (**Supplementary Figure S11**). **(E)** Immunofluorescence was employed to image the localization of NFATc1 in pre-OCs at day 3. Nucleus occupancy of NFATc1 was evaluated by Imaris. Cells with NFATc1 present in the nucleus were counted. Scale bar: 10 μm. Data represent the mean ± SD of three independent experiments. *p < 0.05 ***p < 0.001 ****p < 0.0001.

RANK-positive mononuclear pre-OCs, which are derived from monocyte lineage cells, circulate in the vasculature and migrate to bone (58). Fluid shear stress influences the local migration of circulating immune cells, such as CD4 T cells, neutrophils, and monocytes (59–61). As demonstrated by the above-noted result (**Figure 1E**) and a previous report (62), Piezo1, but little, or no, Piezo2, is expressed by pre-OCs. The functionality of Piezo1 in pre-OCs was examined by siRNA-based loss-of-function assay. The silencing of Piezo1 mRNA by Piezo1-specific siRNA (siPiezo1) in pre-OCs (**Figure 1I**) downmodulated Ca^2+^ influx induced by Yoda1, a chemical agonist of Piezo1, which was not observed in the pre-OCs treated with siControl (**Figure 2J**). The shear flow (20 dyn/cm^2^) created in a microfluidics system caused an influx of Ca^2+^ in pre-OCs. Such shear flow-induced Ca^2+^ influx was, however, abrogated by treating pre-OCs with siPiezo1 (**Figure 2K**). Also, mechanical stress generated by the rocker suppressed OC-genesis-related genes, including the expression of OCSTAMP, MMP9 and ACP5, while mature TRAP+ OC formation and pit formation were downregulated by treating pre-OCs with siPiezo1 (**Figure 2L**; **Supplementary Figure S1g**). These data indicated that Piezo1 expressed in pre-OCs acts as a mechanosensor that downregulates RANKL-induced OC-genesis.

### 3.5 Pharmacological Piezo1 activator inhibited OC-genesis and function

Yoda1 is a chemical agonist that can selectively open Piezo1 and promote intercellular Ca^2+^ to initiate a variety of biological events (63–66). We therefore used Yoda1 to determine if RANKL-induced OC-genesis was regulated by Piezo1. We found that TRAP-positive multinucleated OC formation, as well as bone resorptive activity, were both significantly diminished by Yoda1 administration (**Figure 2A**). In addition, GsMTx4 inhibited Yoda1-induced Ca^2+^ influx in pre-OCs (**Supplementary Figure S1e**). To test the effect of Yoda1 on human OC-genesis, peripheral blood mononuclear cells (PBMC)-derived pre-OCs were employed. Yoda1-mediated Piezo1 activation also inhibited RANKL-mediated human OC-genesis (**Supplementary Figure S2a**). PCR array was used to screen for RANKL-stimulated genes that were impaired by Yoda (**Figure 2B**). Yoda1-mediated suppression of OC-genesis-associated genes (*Ocstamp*, *Ctsk*, *Mmp9*, *Acp5* and *Oscar*) was also confirmed by qPCR (**Supplementary Figure S2b**). Upon stimulation with RANKL, NFATc1, a master TF of OC-genesis (10, 67, 68), translocates from cytoplasm to nucleus, and induces the transcription of genes required for OC-genesis and fusion (69, 70). Yoda1 inhibited the expression of *Nfatc1* gene and its protein during RANKL-induced OC-genesis (**Figures 2C–E** and **Supplementary Figure S5a**). Furthermore, Yoda1 inhibited NFATc1 nuclear localization and disrupted the redistribution of β-actin from the inner to the outer regions, suggesting that Yoda1 impairs osteoclast mobility, preventing fusion with adjacent OCs (**Figure 2E**). These results suggested that the pharmacological activation of Piezo1 caused the downregulation of RANKL-induced OC-genesis in conjunction with the suppression of both *Nfatc1* expression and NFATc1 nuclear translocation.

### 3.6 Piezo1 activation by Yoda1 strongly suppressed Akt phosphorylation.

**Figure 3:**
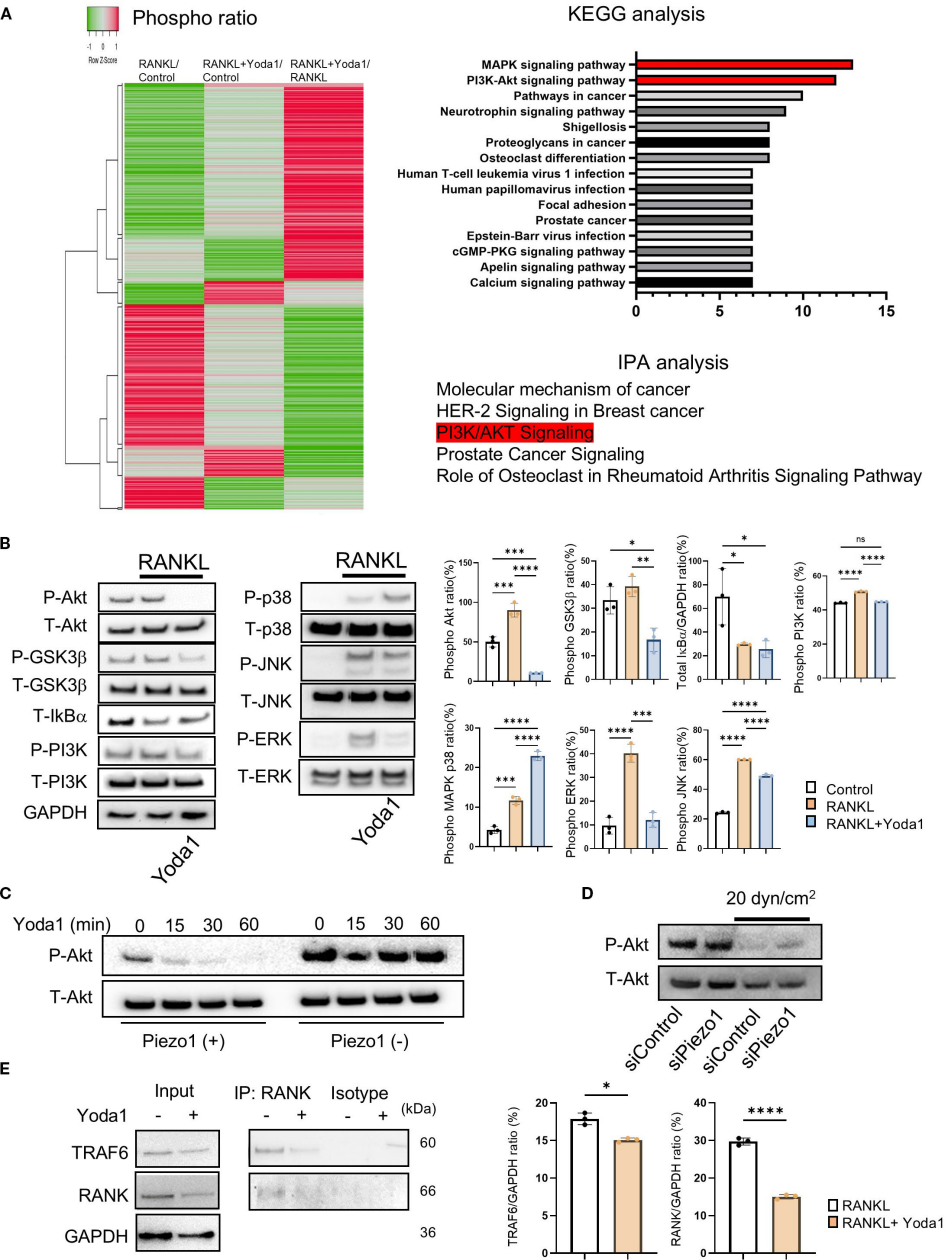
Piezo 1 activation by Yoda1 strongly suppressed Akt phosphorylation. **(A)** Phospho Explorer Antibody Array was performed to determine Yoda1-mediated signaling pathway in pre-OCs. KEGG and IPA were used for bioinformatics analysis. **(B)** Pre-OCs were stimulated with RANKL (10 ng/ml) in the presence or absence of Yoda1 (5 uM) for 30 min to monitor protein phosphorylation, including Akt, GSK-3b, PI3K, p38^MAPK^, ERK, JNK and NF-kB. siRNA-transfected OCs were cultured with shear flow, and samples were collected to monitor Akt phosphorylation by Western blotting. Densitometric analysis was conducted using ImageJ software (Version 1.50). **(C)** Pre-OCs from Piezo1^flox/flox^ mice or Piezo1^LysMΔ^ mice were stimulated with Yoda1 for the indicated time courses, and Akt phosphorylation was assessed by Western blotting. **(D)** Pre-OCs treated with siRNA for Piezo1 or negative control were stimulated with shear stress at 20 dyn/cm^2^, and then Western blotting was preformed to determine Akt phosphorylation. **(E)** Pre-OCs were stimulated with Yoda1 for 30min, and cell lysates were subjected to Co-IP to analyze the interaction between RANK and TRAF6. Representative band images are shown from three independent experiments. Data represent the mean ± SD of three independent experiments. *p < 0.05 **p < 0.01 ***p < 0.001 ****p < 0.0001.

Piezo1 is reported to provoke intracellular signaling activation in numerous cells (37, 71, 72). However, Piezo1-related signaling in OCs is still unknown. To address this question, a phospho antibody array was performed to discover the specific signaling of Piezo1 in pre-OCs *in vitro*. A search of the Kyoto Encyclopedia of Genes and Genomes (KEGG) and Ingenuity^®^ Pathway Analysis (IPA^®^) found PI3K/Akt signaling to be the most likely Piezo1 signaling pathway (**Figure 3A**). Akt is a serine/threonine kinase that plays a critical role in cell survival, growth, and metabolism. It is well known that Akt is closely involved in OC-genesis (73, 74). Akt phosphorylation activates GSK3β, then evokes NFATc1 nuclear translocation during OC formation (75–77). As further confirmation, the inhibition of PI3K/Akt signaling by LY294002, a morpholine-containing chemical compound, resulted in suppressed OC formation and Nfatc1 expression, suggesting that PI3K/Akt signaling is, indeed, involved in OC-genesis (**Supplementary Figure S1f**). In addition, Yoda1 application significantly reduced Akt phosphorylation and moderately inhibited ERK phosphorylation (**Figure 3B**). The inhibitory effect of Yoda1 on Akt phosphorylation in OCs was observed in a time-dependent manner, but it was diminished in Piezo1-deficient OCs (**Figure 3C**; **Supplementary Figure S5b**)4f Moreover, shear flow-mediated Akt dephosphorylation was regulated via Piezo1 (**Figure 3D**; **Supplementary Figure S5b**). Therefore, Akt dephosphorylation induced by Piezo1 activation is associated with OC formation. RANKL/RANK signaling is known to involve the TRAF6/PI3K/Akt pathway in OCs (78). To further clarify the interaction between Piezo1 activation and RANK/TRAF6/PI3K signaling, we examined the effects of Yoda1 on RANK/TRAF6 binding and PI3K phosphorylation. Piezo1 activation by Yoda1 suppressed RANK/TRAF6 binding, which was accompanied by a reduction in PI3K phosphorylation (**Figure 3B, E**). However, unlike the strong suppression observed in Akt phosphorylation (**Figure 3B**), PI3K phosphorylation was only partially inhibited by Yoda1. These findings indicate that Piezo1 activation partially regulates OC-genesis by interfering with RANK/TRAF6/PI3K signaling.

### 3.7 PP2A, not Calcineurin, is involved in Piezo1-induced Akt dephosphorylation in OCs.

**Figure 4:**
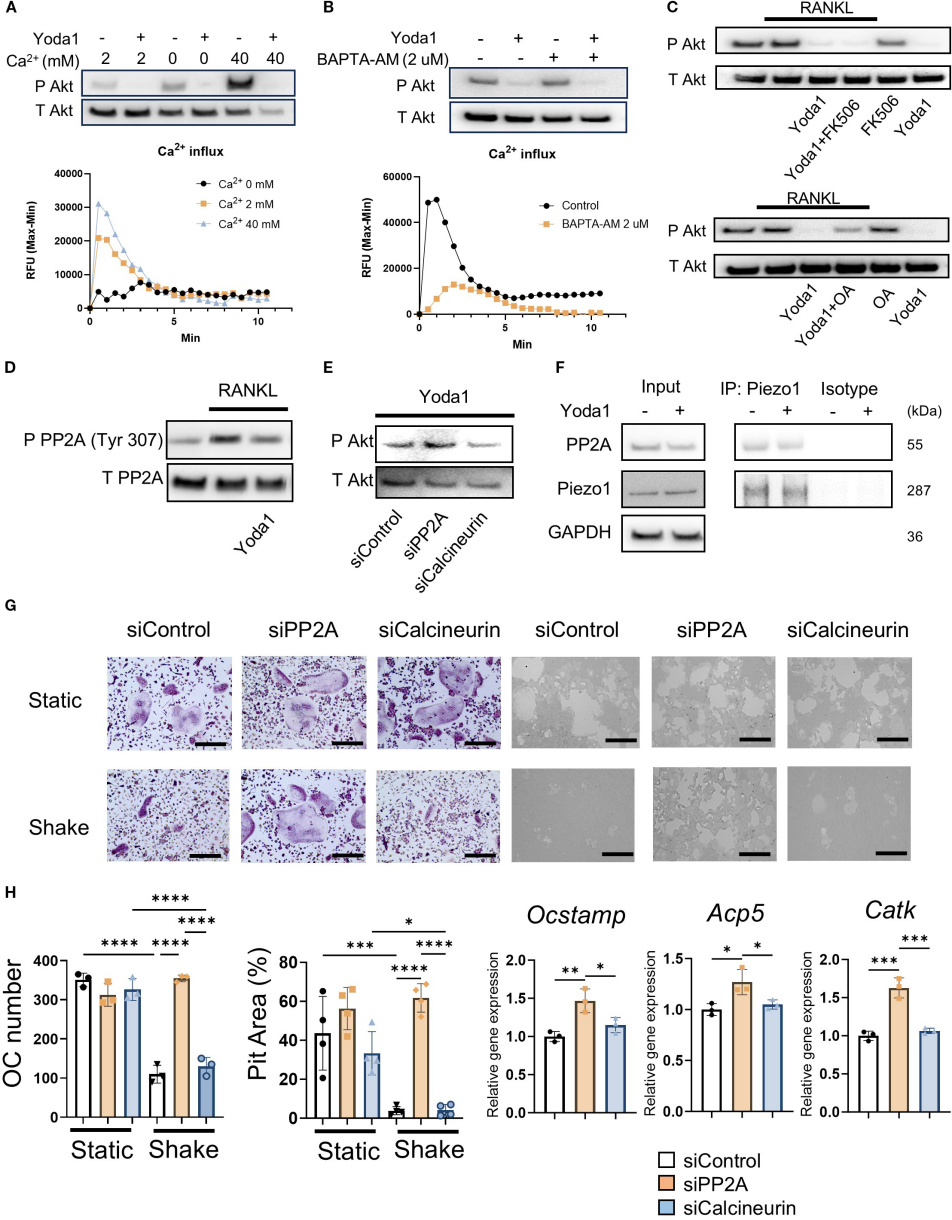
Piezo1 activation in OCs induced PP2A-mediated Akt dephosphorylation in a Ca^2+^-independent manner. **(A)** Pre-OCs were stimulated with Yoda1 in the medium containing 0, 2 or 40 mM Ca^2+^. Akt phosphorylation was evaluated by Western blotting and Ca influx was observed. **(B)** Pre-OCs were preincubated with BAPTA-AM (2uM) constituted in Hanks’ Balanced Salt Solution (HBSS) and 0.1% of Pluronic F-127 for 30 min. After washing with HBSS twice, fresh medium was added followed pre-OCs were stimulated with Yoda1. Akt phosphorylation was evaluated by Western blotting and Ca influx was observed. **(C)** Following preincubation with okadaic acid (protein phosphatase inhibitor) (250 nM) or FK506 (calcineurin inhibitor) (1 uM) for 1 hour, pre-OCs were incubated with Yoda1 (5 uM) for 30 min. Western blotting was performed to detect Akt phosphorylation. **(D)** *Phosphorylation* of the *PP2A* catalytic subunit at Tyr307 was visualized by Western blotting. Densitometric analysis was performed, and data are shown. **(E)** Either siPP2A or siCalcineurin was employed to evaluate Yoda1-mediated Akt dephosphorylation by Western blotting. Densitometric analysis was performed, and data are shown. **(F)** Pre-OCs were stimulated with Yoda1 for 30min, and cell lysates were subjected to Co-IP to analyze the interaction between Piezo1 and PP2A. **(G, H)** To determine the effect of PP2A or calcineurin on mechanical force downregulation of OC-genesis, pre-OCs were transfected with siPP2A, siCalcineurin or siControl, followed by TRAP staining, pit formation assay and qPCR for *Ocstamp, Acp5* and *Catk* expression. Scale bar: 10 μm. Data represent the mean ± SD of three independent experiments. *p < 0.05 **p < 0.01 ***p < 0.001 ****p < 0.0001.

**Figure 5:**
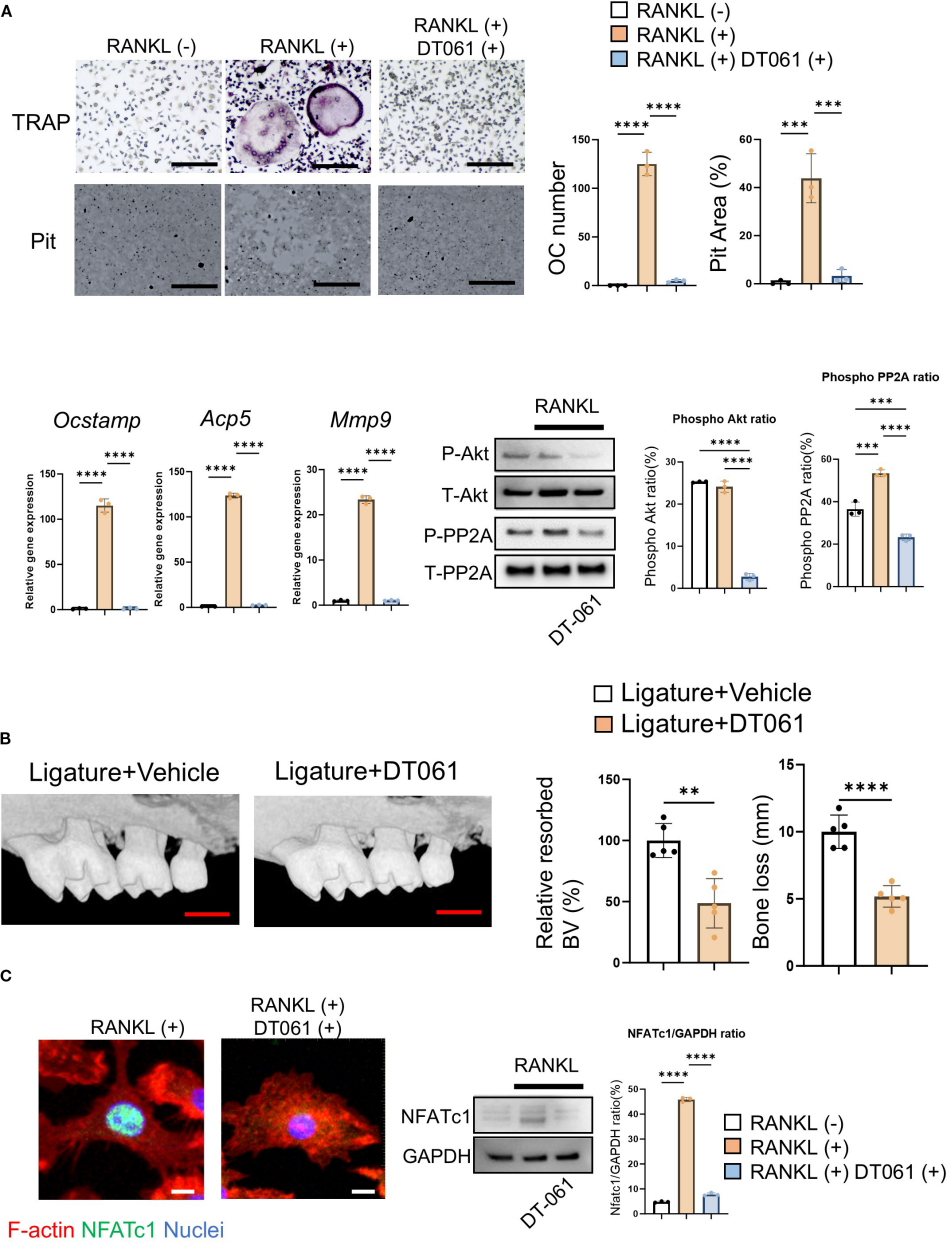
DT-061, PP2A activator, attenuates OC-genesis via Akt signaling in periodontitis. **(A)** DT-061 (5 uM) or vehicle control was added to pre-OCs to analyze OC-related gene expression, including *Ocstamp, Acp5* and *Mmp9*, along with RANKL-mediated Akt phosphorylation. Scale bar: 10 μm **(B)** DT-061 (0.4 mg/kg), or vehicle control (DMSO, 0.86%), was systemically injected into the periodontitis area of mice induced by silk ligation. Scale bar: 1 mm **(C)** DT-061- (5 uM) or vehicle control-mediated NFATc1 protein expression was imaged. Scale bar: 5 μm. Results were presented as the means ± SD. **p < 0.01 ***p < 0.001 ****p < 0.0001.

In the absence of extracellular Ca^2+^, Yoda1 was not able to induce Ca^2+^ influx in pre-OCs, whereas Yoda1 induced Ca^2+^ influx in pre-OCs suspended with Ca^2+^ at 2 mM (normal Ca^2+^ concentration in medium) and 40 mM (high Ca^2+^ concentration representing on the bone surface) (**Figure 4A**; **Supplementary Figure S5c**). BAPTA-AM is mostly used for cell-permeable intercellular Ca^2+^ chelating agent (79, 80). Whereas BAPTA-AM could block Yoda1-induced intercellular Ca^2+^ influx, Yoda1 could induce Akt-dephosphorylation in the presence of BAPTA-AM, suggesting that a Ca^2+^-independent pathway mediates Yoda1-induced Akt dephosphorylation in OCs (**Figure 4D**; **Supplementary Figure S5d**). Akt is regulated by the protein phosphatase family, such as protein phosphatase 2A (PP2A) or protein phosphatase 2B (PP2B), known as calcineurin (81). We demonstrated that okadaic acid, a PP2A inhibitor, but not FK506, a calcineurin inhibitor, could counteract Yoda1-induced dephosphorylation of Akt (**Figures 4C, F**; **Supplementary Figures S5e, d**). Subsequently, as a gain-of-function approach, DT-061, a PP2A activator (82), was employed to elucidate PP2A’s functional role in OC-genesis. We demonstrated that DT-061, through its activation of PP2A, significantly suppressed TRAP+ OC formation, resorption pit formation, and Akt phosphorylation in RANKL-stimulated pre-OCs (**Figure 5A**). RANKL-induced NFATc1 expression was also downregulated by treatment with the PP2A activator DT-061 (**Figure 5C**). We found that Yoda 1 downregulated the induction of PP2A phosphorylation at *Tyr^307^* in RANKL-stimulated pre-OCs (**Figure 4D**; **Supplementary Figure S5g**). It is noteworthy that the PP2A catalytic subunit (PP2Ac) is inactivated by single phosphorylation at *Tyr^307^* residue (83), whereas phosphorylation of *Tyr^127^* and *Tyr^284^* can activate PP2Ac (84). These findings indicate that the activation of Piezo1 can suppress phosphorylation of PP2A at *Tyr^307^* to increase phosphatase activity by PP2A which, in turn, suppresses the phosphorylation of Akt, i.e., Akt dephosphorylation, as well as NFATc1. RNAi-based silencing of PP2A mRNA expression (siPP2A), but not calcineurin (siCalcineurin), resulted in increasing Akt phosphorylation in Yoda1-treated OCs (**Figure 4E** and **Supplementary Figure S5h**). Furthermore, treatment with siPP2A, but not siCalcineurin, prevented shear stress-dependent suppression of RANKL-stimulated OC-genesis, otherwise activated by PP2A, including TRAP-positive OC formation, pit formation, and OC-genesis-related gene expression (**Figures 4G, H**). Collectively, these results suggested that Piezo1-mediated mechanosensing by pre-OCs suppresses RANKL-induced OC-genesis through cell signaling that involves the PP2A/Akt-dephosphorylation pathway toward the suppression of NFATc1, the master TF controlling OC-genesis. To confirm the correlation between Piezo1 and PP2A, a Co-IP assay was performed using an anti-Piezo1 antibody. The results demonstrated that PP2A directly binds to Piezo1, indicating that Piezo1 activation in osteoclasts may directly regulate PP2A-mediated Akt dephosphorylation (**Figure 4F**; **Supplementary Figure S5i**).

### 3.8 Yoda1 administration prevents osteoclastic bone resorption in a mouse model of ligature-induced periodontitis

**Figure 6:**
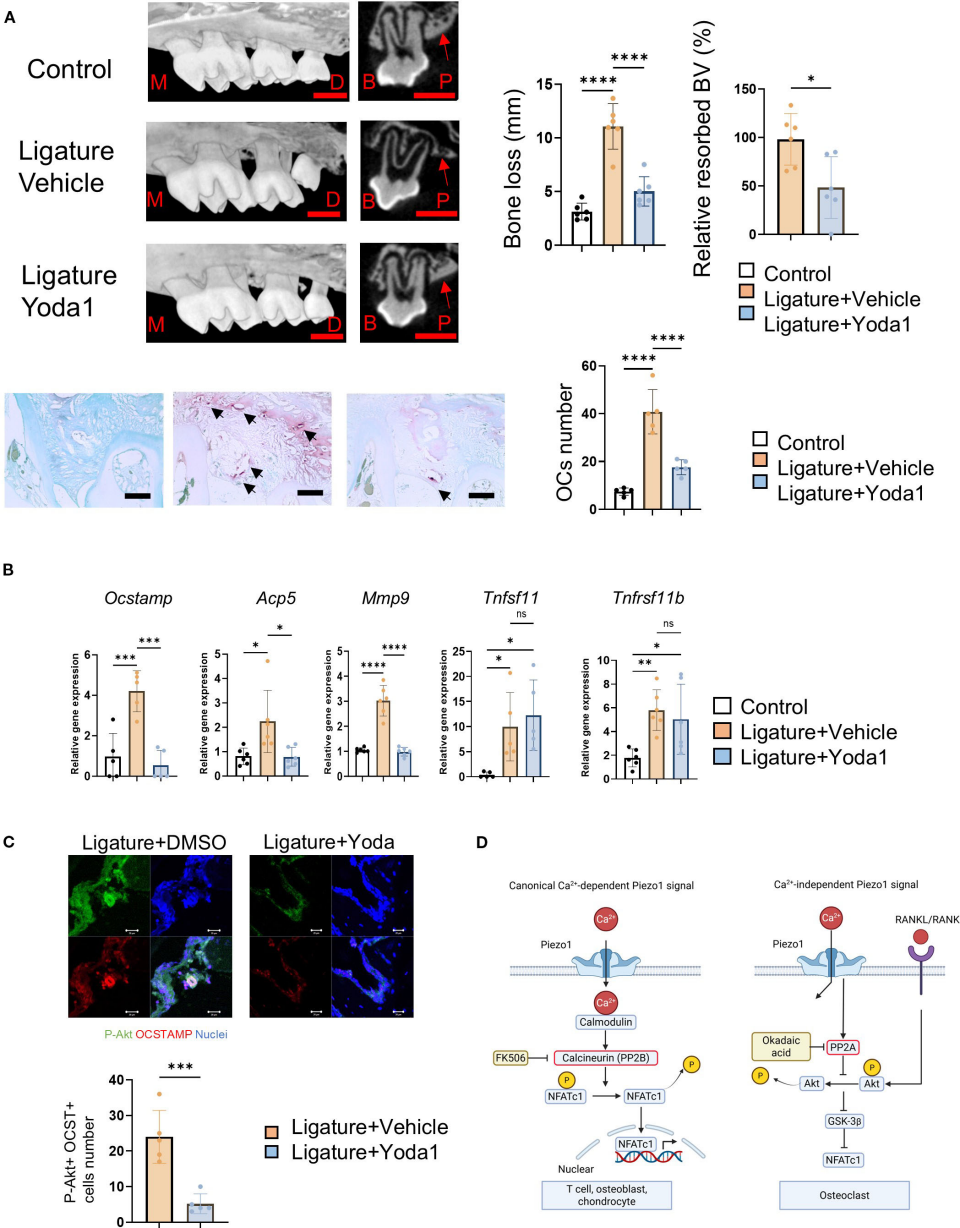
Yoda1 administration prevents osteoclastic bone resorption in murine ligature-induced periodontitis in mice. A 5–0 silk ligature was placed around murine maxillary second molar for 7 days to induce periodontitis. Yoda1 (0.4 mg/kg) or DMSO (as a vehicle control, 0.86%) was administered systemically at day 0, 2, 4 and 6, respectively. **(A)** Bone resorption and TRAP-positive multinucleated OCs were evaluated by micro-CT analysis and TRAP staining. Red scale bar: 1 mm, Black scale bar: 50 μm, M: Mesial side, D: Distal side, B: Buccal side, P: Palatal side. Red arrows indicate the cortical bone of the alveolar bone. Black arrows indicate OCs. **(B)** Gingival tissue samples were harvested for qPCR to measure *Ocstamp*, *Acp5*, *Mmp9*, *Tnfsf11*, *Tnfsf11b* and *Il1b*. **(C)** Frozen tissue sections of mouse model of periodontitis were employed to image phospho-Akt and OC-STAMP double-positive cells in mouse model of periodontitis, and the number of double-positive cells was counted. **(D)** Schematic illustration of canonical Ca^2+^-dependent and Ca^2+^-independent Piezo1 signaling in OCs. scale bar: 20 μm. Results were presented as the means ± SD. *p < 0.05 **p < 0.01 ***p < 0.001 ****p < 0.0001.

Given the decreased shear stress and increased osteoclastic bone resorption observed in periodontitis, we examined the effect of systemic (i.p.) injection of Yoda1 in the mouse ligature-induced periodontitis model. Murine periodontitis was induced by the attachment of a silk ligature at the upper second molar, following previous reports (49, 50, 85). Systemically administered Yoda1 significantly suppressed bone resorption and ligature-induced TRAP-positive OC formation in alveolar bone compared to vehicle control (**Figure 6A**). It also inhibited the mRNA expression of *OCSTAMP*, *ACP5* and *MMP9*, but not RANKL mRNA (*Tnfsf11*) or osteoprotegerin mRNA (*Tnfsf11b*) (**Figure 6B**). Furthermore, the number of phosphorylated Akt-positive OCs increased in mouse alveolar bone (**Figure 6C**).

These results revealed that Piezo1 activation by Yoda1 directly reversed bone resorption in periodontitis by restoring the mechanical stress-signaling in pre-OCs which was attenuated in an inflammation-dependent fashion.

## 4 Discussion

Our findings indicate that Piezo1 plays a dual role in OC-genesis and bone resorption. It negatively regulates OC-genesis by suppressing the expression of key osteoclast marker genes, including *Ocstamp*, *Acp5*, *Mmp9*, *Ctsk*, *Oscar*, and *Nfatc1*. This suppression occurs in the context of healthy periodontal bone through the activation of the negative regulator PP2A, which dephosphorylates Akt within the RANKL-induced Akt/NFATc1 signaling pathway, thereby inhibiting bone resorption. Finally, Piezo1 activation by a chemical agonist Yoda1 could downregulate pathogenically elevated OC-genesis in the alveolar bone of periodontally diseased tissue, suggesting the therapeutic potential of Piezo1 agonist.

It was reported that murine arthritis-associated osteoclastogenic macrophages (AtoMs) comprise the CX_3_CR1^hi^ FoxM1^+^ pre-OCs-containing population in inflamed synovium and that they originate from circulating bone marrow cells (86). Our group also demonstrated that locally produced macrophage migration inhibitory factor (MIF) at the inflammatory bone lytic site is engaged in the chemoattraction of circulating CXCR4^+^ pre-OCs to the inflammatory bone resorption site (22). Accordingly, owing to lower blood flow velocity in periodontitis (29), it is plausible that the diminished shear stress may affect the fate of pre-OCs differentiating into mature OCs. In support of this hypothesis, we observed a significant alteration in blood flow during murine ligature-induced periodontitis, characterized by reduced blood flow in periodontitis-affected tissue compared to healthy control tissue (**Figure 1B**). We found that pre-OCs express a functional Piezo1 mechanosensory ion channel (**Figure 1E**) as a kind of negative rescue factor by the imposition of shear force that otherwise downregulates OC formation via Piezo1 Ca^2+^ ion channel (**Figure 1L**). Thus, it was further hypothesized that Piezo1 may act as a major mechanoreceptor in circulating pre-OCs and that once activated, Piezo1 channels could initiate the PP2A-Akt signaling pathway to downmodulate the expression of genes associated with OC differentiation.

Piezo1 is a key mediator of mechanotransduction in bone cells, including osteoblasts, osteocytes and mesenchymal stem cells (54, 87, 88). It is involved in the differentiation of mesenchymal stem cells into osteoblasts or odontoblasts (46, 54), and is responsible for creating mechanical force and converting it into biochemical signals that regulate cellular responses. In response to mechanical stimuli, Piezo1 channels open, allowing the influx of Ca^2+^ into OBs; this Ca^2+^ influx then triggers a cascade of intracellular signaling events that ultimately lead to bone formation, including, as noted above, activation of ERK or, in our case, Akt cascade (89). Wang et al. reported that the Piezo1/YAP1/collagen pathway is associated with OB maturation *in vivo* and *in vitro* (43). Osteocytes also sense mechanical force through Piezo1, and intracellular signaling occurs in osteocytes through the Piezo1/Akt axis (88) which appears to be transduced by PI3K (90). Our data demonstrates that Piezo1 activation in osteoclasts downregulates Akt signaling (**Figures 3B–D**), while shear stress did not alter Piezo1 expression levels (**Supplementary Figure S1a**). This indicates that Piezo1 in OCs plays a distinct mechanosensory role compared with other bone cell types. Moreover, these findings suggest that the mechanosensory function of Piezo1 in OCs is driven by its activation state rather than by changes in expression. More specifically, based on our study and those of others, mechanosensing via Piezo1 not only promotes osteoblastic bone formation but also inhibits osteoclastic bone resorption through distinctly facilitated Piezo1-mediated cellular signaling pathways (**Figure 3**).

As previously noted, NFATc1 is a master TF controlling OC-genesis. Ligation of RANKL to RANK expressed on pre-OCs elicits cell signals involving the TRAF6/PI3K/Akt axis for induction of NFATc1 nuclear-translocation which, in turn, activates OC-genesis (10, 91). However, Yoda1, the Piezo1 agonist, inhibited NFATc1 expression in pre-OCs stimulated with RANKL (**Figures 2C–E**). Phospho Antibody Array (**Figure 3A**) indicated that Akt plays a key regulatory function in Piezo1-elicited cell signaling for OC-genesis. Indeed, the PI3K/Akt axis plays a crucial role in OC formation (76), whereas interaction of Akt-mediated activation of GSK-3β downmodulates OC formation via inhibition of nuclear translocation of NFATc1 (76, 92, 93). Moreover, Yoda1 only partially inhibited PI3K phosphorylation downstream of RANK/TRAF6 colocalization (**Figure 3B**; **Figure 4F**), whereas Akt phosphorylation was strongly suppressed. Taken together, these findings suggest that Piezo1-mediated signaling primarily suppresses the Akt/NFATc1 axis.

PP2A and PP2B, also known as calcineurin, are protein phosphatases that dephosphorylate specific substrates and play important roles in cell signaling and regulation (94, 95). We demonstrated that Piezo1 activation in OCs promoted PP2A-mediated Akt dephosphorylation (**Figures 4A–C**). Furthermore, we determined that DT-061, a PP2A activator, exerts a crucial preventive effect on OC-mediated bone resorption in murine periodontitis (**Figure 5B**). Myung et al. reported that PP2A inactivation promotes OC-genesis (96). Hyun-Jung et al. also indicated that Dauricine, an isoquinoline alkaloid, decreases OC formation via activation of PP2A (97). These reports and our results strongly suggest that activation of PP2A in OCs can negatively control their differentiation. Furthermore, we discovered, for the first time, that activation of Piezo1 negatively regulates OC-genesis via the PP2A/Akt axis. On the other hand, calcineurin (PP2B) is a Ca^2+^- and calmodulin-dependent serine/threonine protein phosphatase (98). Calcineurin inhibition by FK506 or siRNA was ineffective in dephosphorylating Akt and failed to abrogate shear stress-mediated suppression of OC formation (**Figures 4C, G, H**). Indeed, chemical-based inhibition of calcineurin results in the induction of osteoblastic bone formation (99, 100), but suppression of OC formation (101, 102). In addition, Piezo1 was found to colocalize with PP2A in osteoclasts (**Figure 4F**). Therefore, we concluded that Piezo1-mediated dephosphorylation of Akt depends on PP2A, not calcineurin, in OCs. Moreover, although Piezo1 activation is reported to induce its downstream cell signaling in Ca^2+^ influx-dependent manner (103, 104), we found that the activation of Piezo1 expressed on OCs activates PP2A enzyme in a Ca^2+^-independent fashion (**Figure 4F**).

Ca^2+^ influx is strongly associated with OC-genesis. RANKL/RANK binding allows Ca^2+^ influx, following NFATc1 activation (10). RANKL-mediated OC genesis requires a costimulatory signal characterized by Ca^2+^ influx from ITAM receptors, such as OSCAR and TREM2, triggered by type 3 collagen (11, 12). However, both RANKL and type 3 collagen did not induce Ca^2+^ influx in pre-OCs (**Supplementary Figure S3**). Ionomycin, a Ca^2+^ ionophore, is reported to induce Ca^2+^ influx and OC-genesis (105). Instead, however, ionomycin administration at previously reported concentration (500 nM) increased Ca^2+^ influx, but suppressed OC-genesis (**Supplementary Figures S4a–c**). Thus, it was clear that intracellular calcium influx is not necessarily a positive regulator of OC-genesis.

Ligature-induced periodontitis in mice is a well-established model of periodontitis, as published in our previous reports (49, 106, 107). Here, we demonstrated that systemic Yoda1 application in mice significantly prevented murine periodontal bone loss induced by placement of ligature (**Figure 6A**). Yoda1 is widely used as a specific pharmacological activator of Piezo1 and has applications in the analysis of the bioactivity of Piezo1 in various cells (63, 108, 109). For example, Yoda1 administration in mice bolstered microglial phagocytosis resulting in Aβ clearance in Alzheimer’s disease (110). Yoda1 administration did not alter the body weight of mice; instead, it increased cortical thickness and cancellous bone mass in the distal femur of mice (111). Our results indicated that Yoda1 alone doesn’t affect bone resorption in the control without-ligature group (data not shown). In addition to significantly counteracting bone loss in the mouse model of ligature-induced periodontitis, Yoda1 suppressed gene markers of OC-genesis, including *Ocstamp*, *Acp5* and *Mmp9*, but not *Tnfsf11* and *Tnfsf11b* (**Figure 6B**), and phosphorylated Akt-positive OCs at the alveolar bone surface of murine periodontitis (**Figure 6C**). These results suggest that Yoda1 directly suppressed ligature-induced OC formation *in vivo*.

In summary, we have identified that pre-OCs express functional Piezo1, but not Piezo2, and that mechanical and chemical activation of Piezo1 expressed on pre-OCs downregulates RANKL-primed OC-genesis through Ca^2+^-independent dephosphorylation of Akt by PP2A, rather than the canonical Ca^2+^-dependent Piezo1 pathway reported in various cell types (59, 87, 112). This mechanism ultimately suppresses the expression of NFATc1, a master TF for RANKL-induced OC-genesis (**Figure 6D**). Furthermore, systemic administration of Yoda1, a Piezo1 chemical agonist, can substitute the mechanical stress which was attenuated in the inflamed periodontium of the mice with ligature-induced periodontitis, resulting in the inhibition of local bone resorption mediated by osteoclasts. The feedforward mechanism by Piezo1 chemical agonist that can substitute the mechanical stress lost in the inflammatory bone lytic lesion is anticipated to develop a novel regimen for periodontitis as well as other inflammatory bone lytic diseases such as rheumatoid arthritis and osteoporosis.

## Data Availability

The original contributions presented in the study are included in the article/**Supplementary Material**, further inquiries can be directed to the corresponding author/s.
