# Supplementary material for: Piezo1 protects against inflammatory bone loss via a unique Ca2+-independent mechanism in osteoclasts
Source: Front Immunol. 2025 Sep 25;16:1661538. doi: 10.3389/fimmu.2025.1661538 (PMC12507952; doi:10.3389/fimmu.2025.1661538)
Supplement: Supplementary file 1 [file Table1.docx]

Title: **Piezo1 Protects Against Inflammatory Bone Loss via a Unique Ca²⁺-Independent Mechanism in Osteoclasts**

**Supplementary information**


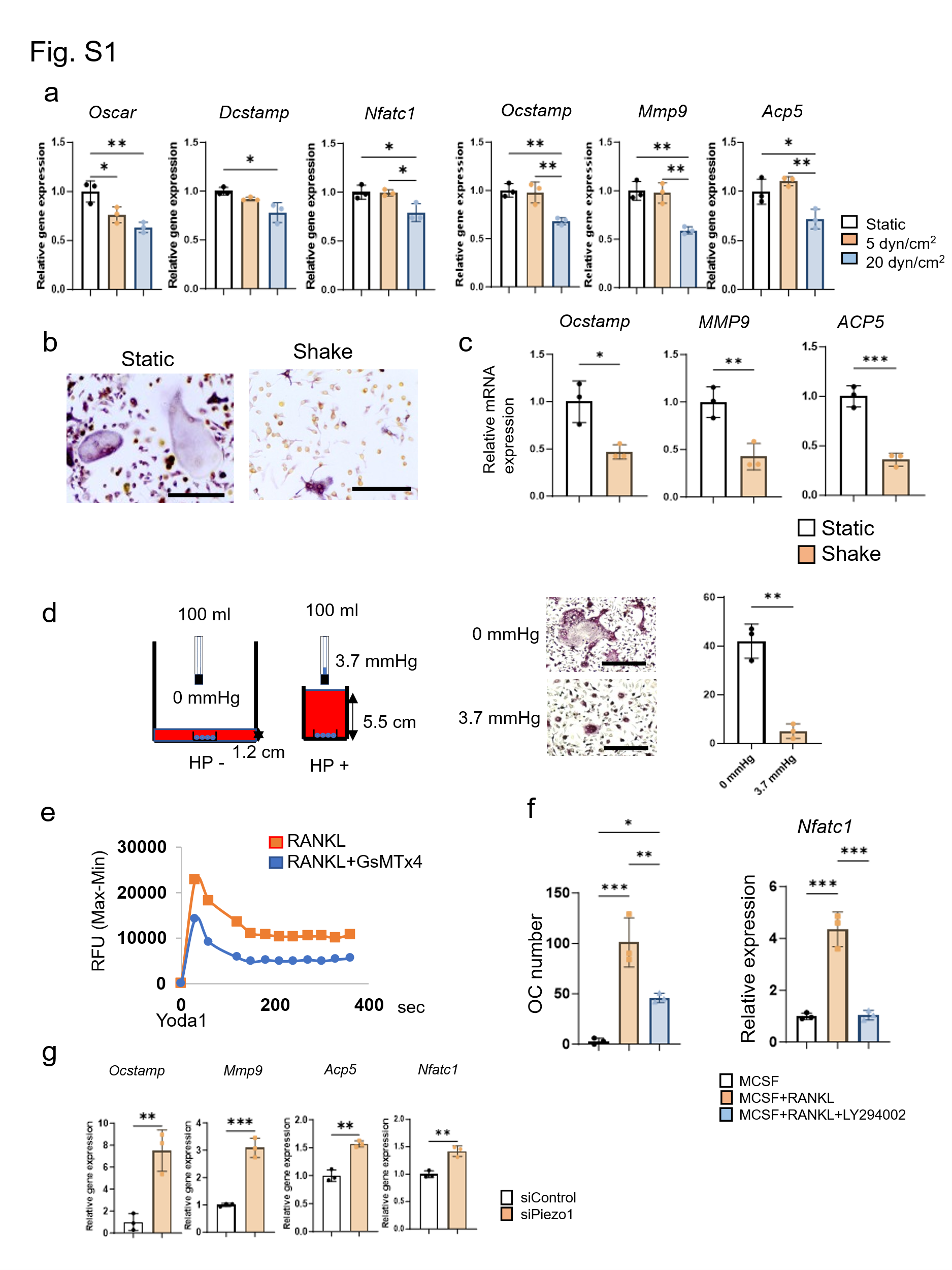


Figure S1. Shear stress generated by shaking inhibits OC-genesis, and GsMT-x4, Piezo1 inhibitor, suppresses Ca^2+^ influx mediated by Yoda1.

(a) RANKL (10 ng/ml)-mediated OC-genesis with or without shear stress at 5 or 20 dyn/cm^2^ by the ibidi pump system was evaluated by qPCR to monitor *OC-associated gene* expression. (b and c) Pre-OCs were subjected to mechanical loading by rocker (15°, 30 rpm) to determine OC-genesis. Scale bar: 10 μm (d) Pre-OCs were cultured under either hydrostatic pressure or atmospheric pressure by using beakers of different sizes but filled with the same volume of medium to assess their effects on OC-genesis. Scale bar: 10 μm (e) Yoda1-induced Ca^2+^ influx in pre-OCs with or without GsMT-x4 (1 uM) was measured. (f) Using LY294002 as an Akt inhibitor, TRAP staining and Nfatc1 expression were each analyzed to confirm the importance of Akt signaling in OC-genesis. (g) The siRNA-treated pre-OCs were stimulated with shear flow by shaking for 2 days. *Ocstamp, Mmp9*, *Acp5* and *Nfatc1* expression was analyzed by qPCR.Data represent the mean ± SD of three independent experiments. * p < 0.05 ** p < 0.01 *** p < 0.001

**
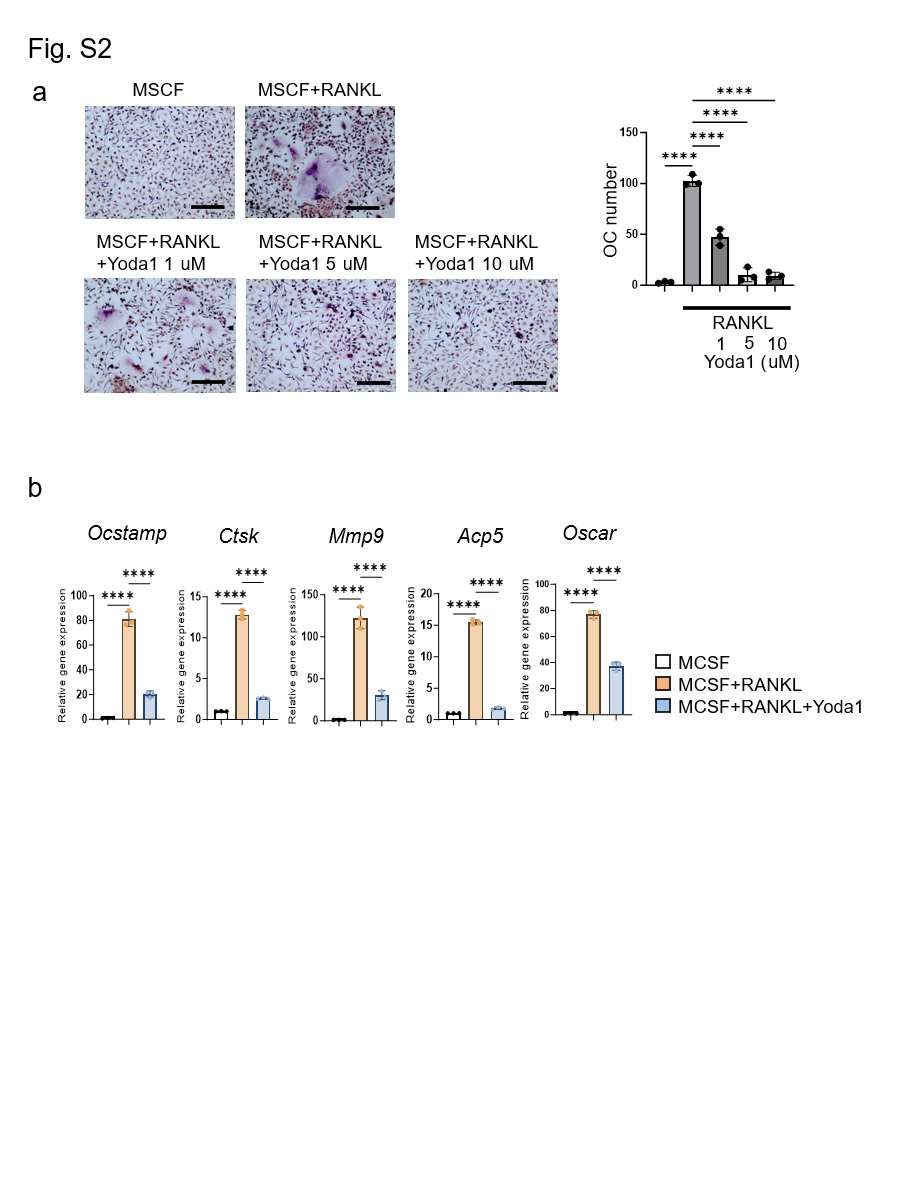
**

Figure S2. Piezo1 activation also inhibits human OC-genesis.

(a) Yoda1 (1, 5, 10 uM) was applied to RANKL-primed human pre-OCs. TRAP- positive multinucleated OCs were counted. Scale bar: 10 μm (b) OC-related gene expression in human pre-OCs with or without Yoda1 stimulation. Data represent the mean ± SD of three independent experiments. Results were presented as the means ± SD. **** p < 0.0001


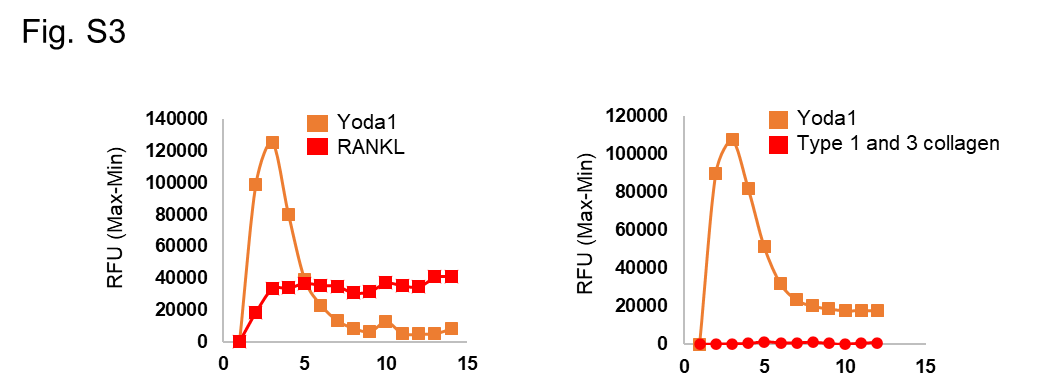


Figure S3. RANKL or either type-1 or -3 collagen does not induce Ca^2+^ influx in OCs. Pre-OCs were labeled with Fluo-8 NW; then Ca^2+^ influx was detected by a FilterMax F5 Microplate Reader.


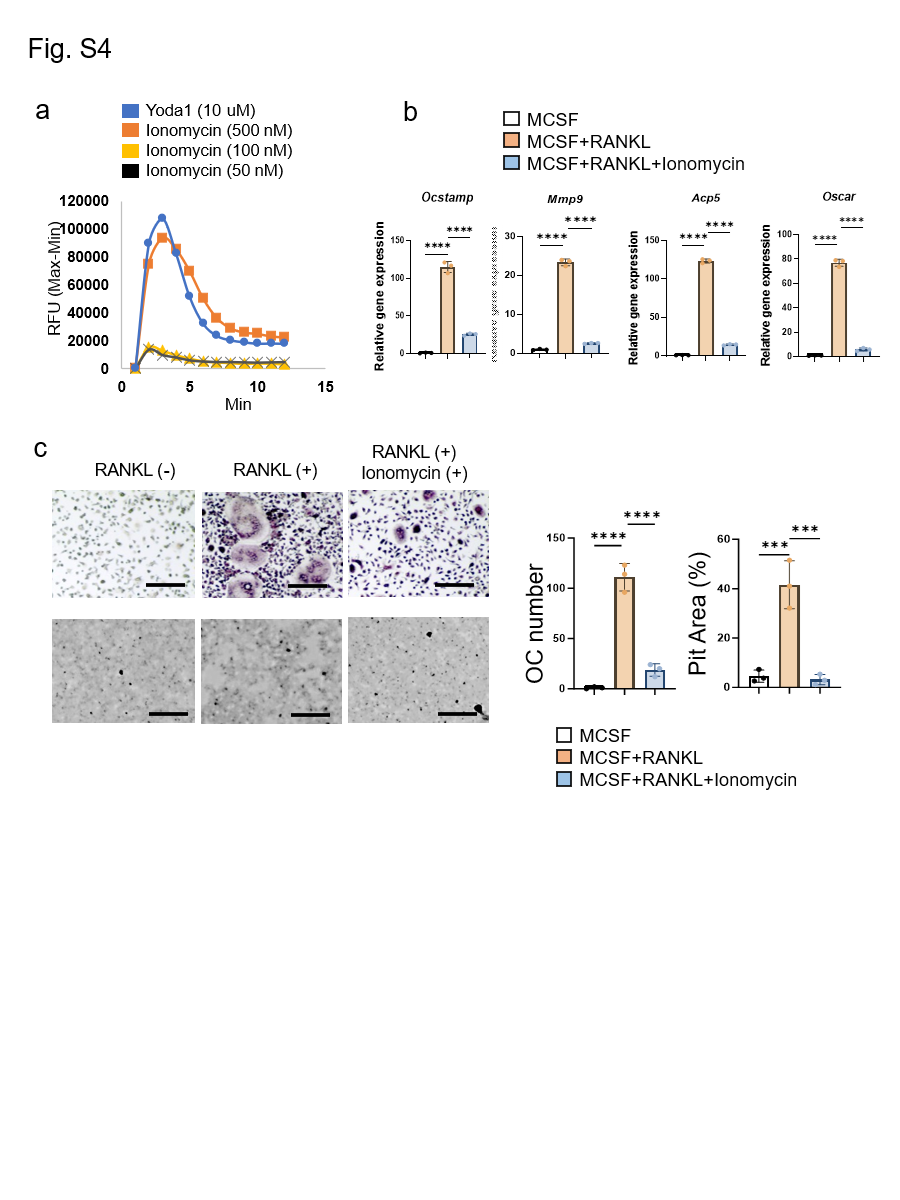


Figure S4. Ionomycin induces Ca^2+^ influx, but inhibits OC-genesis.

(a) Pre-OCs were treated with 500, 100 or 50 nM of Ionomycin to measure Ca^2+^ influx. (b) Ionomycin (100 nM) was applied to RANKL-mediated OCs to investigate the level of OC-related gene expression, including *Ocstamp, Mmp9, Acp5*, and *Oscar*. (c) TRAP staining was conducted to evaluate Ionomycin (100 nM)-mediated OC-genesis. Scale bar: 10 μm

Data represent the mean ± SD of three independent experiments. *** p < 0.01 **** p < 0.0001


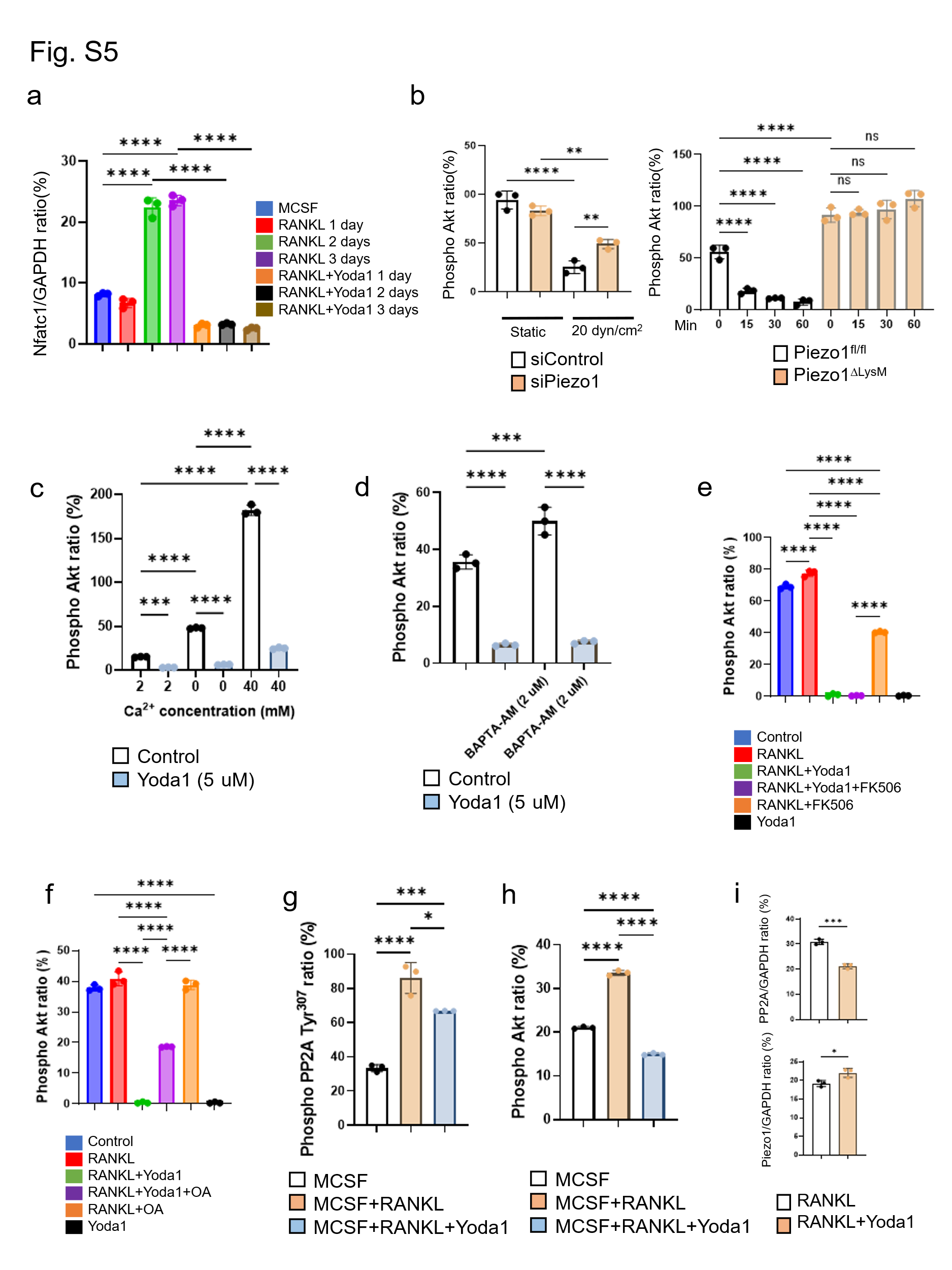


Figure S5. Densitometric analysis for Western blotting and Ca^2+^ influx measurement

(a-i) Densitometric analysis of Western blotting in Fig. 2d, 3c,d, 4a, b, c, d, e and f were conducted using ImageJ software (Version 1.50). Data represent the mean ± SD of three independent experiments. ** p < 0.001 **** p < 0.0001
